# Therapeutic application of regeneration-associated cells: a novel source of regenerative medicine

**DOI:** 10.1186/s13287-023-03428-y

**Published:** 2023-08-02

**Authors:** Amankeldi A. Salybekov, Mehdi Hassanpour, Shuzo Kobayashi, Takayuki Asahara

**Affiliations:** 1 Kidney Disease and Transplant Center, Shonan Kamakura General Hospital, Kamakura, Japan; 2 Shonan Research Institute of Innovative Medicine, Shonan Kamakura General Hospital, Kamakura, Japan

**Keywords:** Regeneration-associated cells, Endothelial progenitor cells, Cell therapy, Regenerative medicine

## Abstract

Chronic diseases with comorbidities or associated risk factors may impair the function of regenerative cells and the regenerative microenvironment. Following this consideration, the vasculogenic conditioning culture (VCC) method was developed to boost the regenerative microenvironment to achieve regeneration-associated cells (RACs), which contain vasculogenic endothelial progenitor cells (EPCs) and anti-inflammatory/anti-immunity cells. Preclinical and clinical studies demonstrate that RAC transplantation is a safe and convenient cell population for promoting ischemic tissue recovery based on its strong vasculogenicity and functionality. The outputs of the scientific reports reviewed in the present study shed light on the fact that RAC transplantation is efficient in curing various diseases. Here, we compactly highlight the universal features of RACs and the latest progress in their translation toward clinics.

## Background

Following the isolation of endothelial progenitor cells (EPCs) [1, 2], autologous total mononuclear cells (MNCs) freshly isolated from bone marrow (BM) or peripheral blood (PB) have been examined for clinical ischemic cell therapy in patients with severe cardiac ischemia or peripheral vascular diseases [3]. These initial clinical experiences indicate the safety, feasibility, and effectiveness of cell-based therapies for vascular regeneration [4]. However, translational and clinical trials have shown insufficient or contradictory effectiveness in ischemic disease recovery [5–7].

PB or BM-derived MNCs are composed mainly of hematopoietic lineage cells, including the majority of lymphoid cells and myeloid monocytes, and less of stem/progenitor cells, such as hematopoietic stem/progenitor cells (HSPCs), EPCs, and other mesenchymal stem cells (MSCs) [8]. According to the most recent publications, EPCs are CD31, CD144, kinase insert domain receptor (KDR), and CD133-expressing cells with distinct angiogenic and fascinating immunosuppressive functions [9, 10]. Also, the anti-inflammatory functions of EPCs have been well documented and are mediated by the tumor necrosis factor-α (TNF-α)-TNFR2 axis in EPC immunoregulatory functions [11, 12]. The scarcity of EPCs in the MNC population is one of the main reasons for failing to make the constant and potent contributions in clinical cases. The enriched EPCs, such as CD34- or CD133-positive cells, populate less than 0.01% of PB-MNCs and 0.1% of BM-MNCs, while the frequency of colony-forming EPCs is 0.005% in PB-MNCs [13].

However, referring to the finding of the initial EPC publication that co-cultures of CD34^+^ cells with CD34^+^ depleted MNCs significantly increased proliferation rate and tube-like structure formation rather than CD34^+^ cells culturing alone [1], Kwon et al. [14] disclosed that CD34^+^ cells cocultured with T cells greatly accelerated formation of primitive EPC colony, whereas coculturing with myeloid cells promoted definitive EPC colony formation in an in vitro assay, indicating that regenerative signals derived from myeloid or lymphoid cell subsets may promote EPC vasculogenic potential. Lee et al. [15] demonstrated that the optimal generation of endothelial colony forming cells (ECFCs) from CD34^+^ EPCs requires the presence of accessory CD34-negative MNCs secreting angiogenic cytokines.

**Fig. 1:**
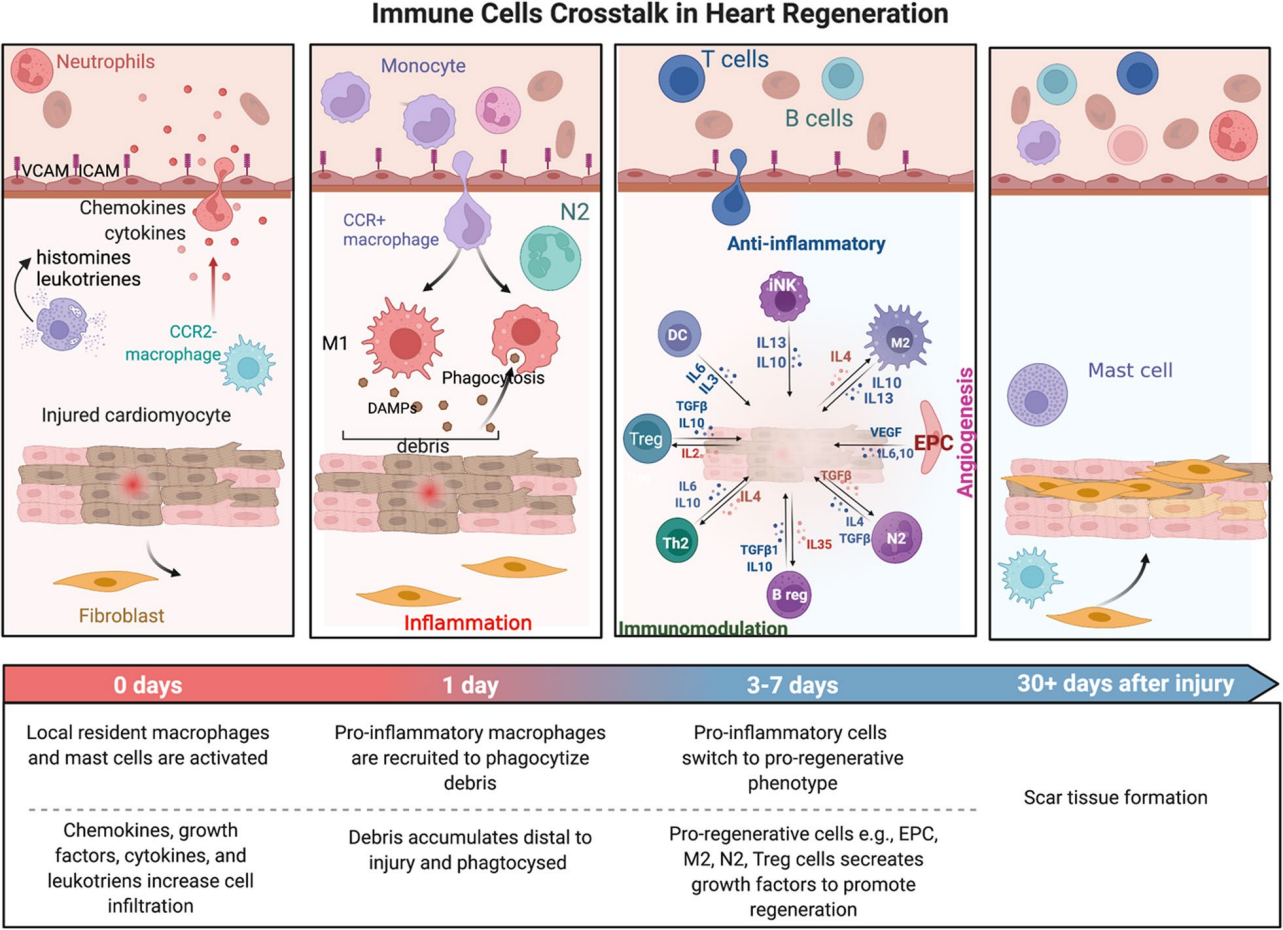
Immune cells crosstalk in heart regeneration. Figure created with BioRender.com

Ischemic injury mobilizes a diverse repertoire of innate and adaptive immune cells. In the inflammatory phase, the abundant number of dying cells secrete chemokines, interleukins, leukotrienes, and growth factors to induce the large-scale production and recruitment of neutrophils, monocytes, and other cells, mainly from HSPCs in the BM (Fig. 1) [16, 17]. In the reparative phase of injuries, pro-inflammatory cells become polarized toward the type 2 macrophage (M2) and type 2 T helper (Th2) phenotypes in response to various environmental stimuli and increase the abundance of regulatory T cells (Tregs) and regulatory B cells (Bregs) (Fig. 1) [18, 19]. In addition, dendritic cells (DCs), natural killer cells (NKs), and forkhead box protein P3+ (FOXP3+)/CD4^+^ T cells begin to secrete interleukin-13 (IL-13), IL-10, transforming growth factor-β (TGF-β), and IL-4 to accelerate monocyte/macrophage plasticity and EPC differentiation (Fig. 1) [14, 20]. Following consideration of the above-mentioned limitations in EPC culture, enrichment, and ischemia tissue immune cells crosstalk, the concept of regeneration-associated cells (RACs) has been suggested to enhance the quality and quantity of vasculogenesis-related cells, i.e., EPCs, anti-inflammation M2, and immune-modulatory Tregs [21]. This article highlights recent advances in cell conditioning methods for the therapeutic efficacy of RAC transplantation in a variety of clinical settings, providing a practical option for cell sources.

## Lessons learned from tumor-associated cells

**Fig. 2:**
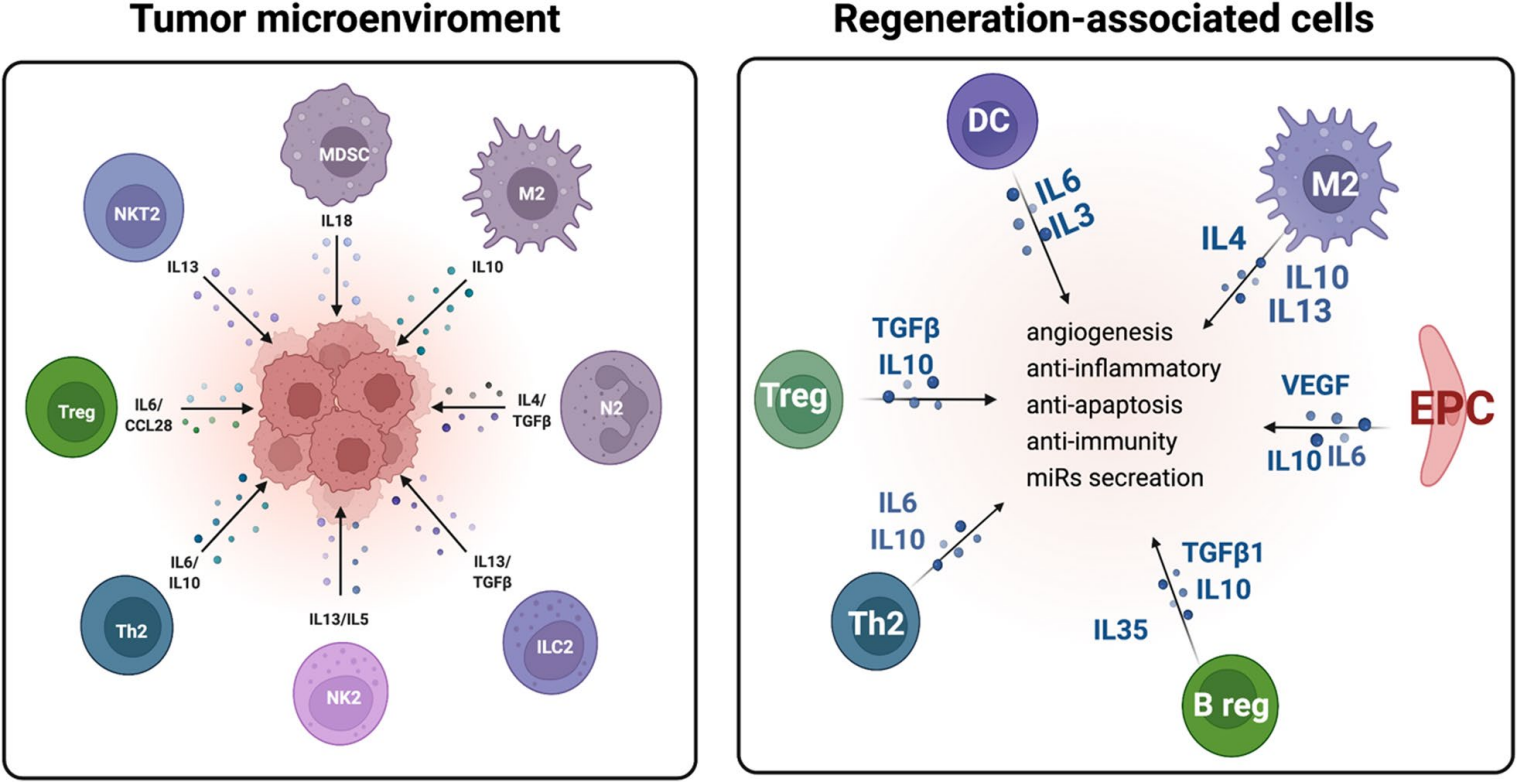
Similarity of tumor-associated cells and regeneration-associated cells. Figure created with BioRender.com

Accumulating evidence suggests that tumor-associated cells (TACs) play an essential role in tumor *vascularization* and evasion of *immune surveillance*. The TAC term groups various cell types that significantly contribute to tumor expansion, also known as tumor-associated macrophages (TAMs), tumor-associated neutrophils (TANs), T and B cells, etc. [22]. Tumor cells constantly interact with the surrounding microenvironment. Recent studies have depicted that the crosstalk between different hematopoietic cells (such as myeloid, lymphoid, and EPCs) and tumor cells in the tumor microenvironment plays an essential role in tumor progression [23, 24]. It has been assessed that almost every tumor type produces chemokines to attract cell trafficking into the solid tumor microenvironment via secreting chemokine (C–C motif) ligand 2 (CCL2), CCL3, CCL5, CCL7, CXCL8 (IL-8), macrophage inflammatory protein 1α (MIP-1α)/CCL3, and human granulocyte chemotactic protein-2 (huGCP-2/CXCL6) [25, 26]. Infiltrated immune cells shift their phenotypes from anti-tumor, also known as pro-inflammatory phenotypes, to pro-tumor phenotypes because tumor cells increase interleukins and chemokines such as IL-2, IL-4, IL-6, IL-7, IL-10, IL-12, and CXCL12 concentrations (Fig. 2) [27–29]. A great example is tumor milieu M2-like macrophages; the latter are by Th2-derived cytokines such as IL-4, IL-10, IL-13, TGF-β, or prostaglandin E2 (PGE2) [30, 31]. They are also known as “repair” or “fix” macrophages as they promote *tissue repair* via *immune tolerance* and *tissue remodeling*, *debris scavenging*, and *immune modulation*. When it comes to cancer, M2-like macrophages support angiogenesis by secreting adrenomedullin and VEGFs and express immunosuppressive molecules such as IL-10, programmed cell death ligand 1 (PD-L1), and TGF-β, favoring tumor growth [30]. They are regarded as “friends” by cancer cells. This lesson from cancer immunology is similar to the concept of the RACs. Under vasculogenic conditioning culture (VCC), naïve PB-MNCs with pro-inflammatory phenotypes shift to pro-regenerative phenotype cells such as vasculogenic EPCs, alternatively activated M2 macrophages, and immunomodulatory lymphocytes, e.g., Tregs and Bregs [32]. This parallel between TACs and RACs has a common point: enhancing angiogenesis, anti-inflammation, and immune modulation effects. RACs as a concept are discussed below (Fig. 2).

## Lessons learned from mesenchymal stem cells

The biological function of RACs is also similar to that of MSCs. MSCs have been proposed as patient-specific drugstores for injured tissue, and they have been scientifically and clinically appealing as sources for cell therapy against a variety of diseases [33]. MSC was originally believed to be a kind of cell population that could differentiate into mesenchymal tissue cells but is now known as a sort of functional cell responding to local environmental stimuli with a myriad of beneficial interventions [34]. The primary trophic property of MSCs is the secretion of growth factors and other chemokines to induce cell proliferation and angiogenesis [35]. Furthermore, through paracrine factors, MSCs assist and modulate the regenerative environment via anti-inflammatory and immunomodulatory mechanisms. In response to inflammatory molecules such as IL-1, IL-2, IL-12, TNF-α, and interferon gamma (IFN-γ), MSCs secrete a variety of growth factors and anti-inflammatory factors such as TGF-β1, hepatocyte growth factor (HGF), stromal cell-derived factor 1 (SDF-1), PGE2, nitric oxide (NO), indoleamine 2,3-dioxygenase, IL-4, IL-6, IL-10, IL-1 receptor antagonist, and soluble TNF-α receptor and maintain complex feedback mechanisms among the many types of immune cells, including monocytes/macrophages, DCs, T helper cells, and NK cells [34].

The availability and versatility of MSCs make them an effective treatment option for a wide variety of clinical pathologies through their angiogenic, anti-inflammatory, and immunomodulatory functions. This strategy and development encourage the application of RACs for future medical contributions to various diseases.

## Regeneration-associated cells as a concept

Over the last two decades, clinical trials using BM-MNCs or purified CD34^+^ and CD133^+^ cells have been performed to cure various cardiovascular severe ischemic patients with modest results [36–38]. Perhaps this may couple with qualitative and quantitative measures, along with crosstalk impairment of EPC with hematopoietic stem cell (HSC) subsets, in patients with concomitant diseases such as diabetes mellitus, hypertension, atherosclerosis, and hyperlipidemia, along with risk-associated factors [39–42]. Furthermore, the scarcity of EPCs in the PB and mobilization barriers in diabetic patients make it difficult to collect enough cells for therapeutic application in clinical settings [39]. Following these considerations, a vasculogenic ex vivo culture system to educate mononuclear cells under vasculogenic signaling to increase regenerative cells qualitatively and quantitatively for tissue regeneration has been developed.

A seminal EPC study found that co-cultures of CD34^+^-depleted MNCs with CD34^+^ cells increased the proliferation rate and tube formation properties in the fibronectin-coated plate significantly more than CD34^+^ cells culturing alone [1]. Subsequently, studies also implied that crosstalk between hematopoietic cells and EPCs plays an important role in terms of EPC differentiation. Therefore, T cells significantly accelerated primitive or small EPC-colony forming units (CFUs), whereas macrophages and megakaryocytes solitary promoted definitive or large EPC-CFU in an in vitro assay in healthy human subjects, indicating that regenerative signals derived from myeloid or lymphoid cell subsets may cause vasculogenic or definitive EPC-CFU maturation [43]. Strikingly, under VCC, naïve PB-MNC phenotypes converted from pro-inflammatory (primitive EPC cells, M1 monocytes/ macrophages (CD192^+^), Th1 (CD4^+^/INF-g^+^/IL4^−^), NK cells (CD56^+^), and B-cells (CD19^+^), etc.) to anti-inflammatory polarized regenerative cells such as definitive or vasculogenic EPC, M2*ϕ* (CD206^+^), T2 (CD4^+^/INF-g^−^/IL4^+^), Tregs (CD4^+^/CD25^+^/FOXP3^+^), Bregs (CD19^+^TIM-1^+^), and DCs [44–47]. These educated or regenerative conditioning-induced PB-MNCs represent RACs with several important regeneration properties, such as immune-modulatory, anti-inflammatory, and strong vasculogenic effects, which have been demonstrated in in vivo experiments in various species [46–49].

The aforementioned characteristics of RACs expand their clinical application ranges, in particular: (i) this product can be transplanted within one week after the start of culture; (ii) the amount of blood collected is small; (iii) it is an autologous cell, so there is little risk of immune rejection; and (iv) it has many clinical application advantages, such as no need for facilities or any special equipment for culture. Below, we will focus on RACs' application to cardiovascular ischemic diseases, nonunion bone fractures, diabetic wound healing, and other diseases (Table 1).

**Table 1:** RACs application on various ischemic and non-ischemic diseases

Condition	Model	Cellular outcomes	General outcomes	Refs
IHD	Rat	Increase EPCs populations Overexpression of VEGF-b, Nkx2-5, GATA-4, and c-kit Decrease in cell infiltration Mobilization of CPCs	Improved transplantation efficacy Preserve cardiac function and fraction shortening Reduce interstitial left ventricular fibrosis Induction of angiogenesis, anti-inflammatory functions, and cardiomyogenesis	[46, 52]
Stroke	Nude mice	Increase VEGF positive cells Increase VEGF and IL-10 Increase EPCs populations	Enhanced cerebral blood supply Decrease neuro-inflammation Reduce the infarct lesion Redevelopment of neurovascular networks	[65, 66]
PAD	Human	Increase angiogenic EPCs populations, CD206^+^ and CD3^+^ and decreased CD14 and CCR2 cells	Accelerate wound healing Increase vascular perfusion and SPP scale Decrease VAS scale	[79, 80]
Ischemic wound healing	Murine and porcine	Increase angiogenic EPCs populations Induce collagen synthesis and maturation Overexpression of FGF, VEGF, IL-4, IL-6, TGF-β and IL-10 Reduction in PGC-1 α and Notch proteins	Enhanced wound closure Improve vasculogenesis Increase anti-inflammatory and immunosuppresive properties in the wound microenvironment,	[84–86]
ischemia/reperfusion kidney injury	Mice	Increase angiogenic EPCs populations Increase paracrine activities Increase CD34^+^/CD133^+^ and CD206^+^ cells	Upgrade renal function and decrease histopathological injury Induce anti-inflammatory functions Decrease BUN and Cr	[53]
Bone disorders	Rat, Human	Differentiation of CD34^+^ cells to osteoblast cells Increase angiogenic EPCs Increased CD34^+^ and CD206^+^ cells Induce synthesis of type I collagen Decrease polymorphonuclear cells infiltration	Enhance fracture healing Increase vasculogenesis anti-inflammatory functions Accelerate wound healing Recovery of the osseous curing process	[95, 102]
Irradiated salivary gland		Increase EPC fraction and anti-inflammatory M2 macrophages Overexpression of IL-10 and VEGF	Recovery of saliva secretion Anti-inflammatory and angiogenesis functions Increase vasculagenesis	[104]

IHD—Ischemic heart diseases; CPCs—cardiac progenitor cells; PAD—peripheral arterial disease; SPP—skin perfusion pressure; VAS—visual analog scale; PGC-1α—peroxisome proliferator-activated receptor gamma coactivator 1 alpha

## RAC therapy for ischemic heart diseases

Emerging data have confirmed that following ischemic injury, M1 macrophages (CCR2^+^) accumulate in tissue to detect endogenous danger signals released by necrotic cell debris via toll-like receptors or via classical antigen-presenting cells like DCs [50]. During the reparative phase, these changes orchestrate adaptive immune response activation immediately after ischemic injury via DCs’ presentation of antigens by major histocompatibility complex (MHC) class I to CD8^+^ cells (cytotoxic or killer T cells) or MHC class II to CD4^+^ cells (helper T cells) [18, 51]. Accordingly, RAC transplantation is crucial to accelerate the switch from a pro-inflammatory milieu to one with anti-inflammatory characteristics and regenerative goals. Recently, Salybekov et al. have demonstrated that RACs, fortified with vasculogenic EPCs and M2 macrophages, improved transplantation efficacy and recovery of ischemic myocardium through induction of angiogenesis, anti-inflammatory functions, and cardiomyogenesis [46, 52]. Excitingly, this angiogenic circumstance could nurture PB-MNCs to increase EPCs (30-fold) and populations accountable for tissue regeneration [53]. Recently, the reciprocal actions concerning EPCs, anti-inflammatory monocytes/macrophages, and T lymphocytes have been well elucidated by a large body of scientific evidence [20, 54]. With reference to these documents, it could be concluded that phenotypic modifications of PB-MNCs in an angiogenic culture microenvironment are sponsored by cross-coupling between EPCs, monocytes/macrophages, and lymphocytes, which are responsible for cell phenotype conversions in ischemic and regenerative microenvironments. These investigations examined the potential of RACs to improve angiogenesis, wound healing efficiency, and organ function recovery [53, 55]. Previous studies reported that after hypoxic cardiomyocyte injury, neutrophils are first recruited to the margin area of the myocardium over 12 h, peaking at day three accompanied by granulocytes, causing a dire predicament, namely a cytokine storm followed by pro-inflammatory monocyte/macrophage incursion after approximately 6 days post-ischemic injury. The excessive influx of pro-inflammatory cells increases matrix metalloproteinase-9 (MMP-9) and inducible nitric oxide synthase (iNOS) activity in the ischemic zone, leading to left ventricular thinning, increased myocardial disability, deteriorating heart function, and a higher mortality rate [56]. In this regard, it has previously been reported that RAC transplantation to ischemic zones led to a decrease in the neutrophil-rich area in the infarcted myocardium. [46]. In recent clinical trials, PB/BM-MNC transplantation did not repair myocardial function implanted into the ischemia-related vessel of MI subjects [57]. Leuschner et al. showed that PB-MNC-transplanted animals presented extension of left ventricular scarring, interstitial fibrosis, and left ventricular wall thickness reduction [58]. These findings could be a result of the fact that 20 percent of PB-MNCs consist of monocytes/ M1 cells, which increase the number of pro-inflammatory monocytes/macrophages in the infarcted zone and result in the expansion of the inflammatory process by means of CCR2 and CX3CR1. However, RAC-transplanted animals exhibit preservation of the infarcted tissues resulting from CD206^+^ cell mobilization [58]. Salybekov et al. transplanted a limited number of RACs to infarcted myocardium and demonstrated the efficacy of RACs for tissue repair and heart function restoration [46]. To further confirm, it has been shown that *FOXP3* is overexpressed in RACs compared to PB-MNCs, which is the reason for the initiation of immune tolerance by Treg function in RACs [20]. Following RACs transplantation, cardiomyogenesis is observed in myocardial ischemic sites. Transcriptomics of RACs showed overexpression of *VEGF-b* and genes involved in early cardiac differentiation (*Nkx2-5*, *GATA-4*, and *c-kit*), a decrease in infiltrated cell numbers, proposing that RACs convert the phenotype of penetrated cells from a pro-inflammatory to an anti-inflammatory state [46, 59]. It is worth noting that RACs could enhance the mobilization of local cardiac progenitor cells (CPCs) into the infarcted area via modifying the VEGF-b/SDF-1 axis, which needs to be concisely studied. According to the previously published report, paracrine activities of RACs induce myocardiogenesis and angiogenesis [45].

RAC-derived extracellular vesicles (RAC-EVs) are another intriguing weapon of these cells. We recently investigated the therapeutic efficiency of RAC-EVs compared with MSC-derived extracellular vesicles (MSC-EVs) in the context of myocardial infarction. Based on our findings, repetitive injection of 5*10^5^ RACs-EVs significantly improved cardiac function, such as the ejection fraction index and mitral regurgitation. Mechanistically, transplanted RAC-EVs deliver various angiogenic factors, also known as angio-miRNAs such as miR-126-3p, -126-5p, -195-3p, -29c-3p, -15b-5p, -195-5p, -200b-5p, -146a-3p, and -146b-5p), anti-fibrosis (via miR-133 and -29-3p), anti-inflammation (via miR-10a-3p, -21-5p, 24-3p, and -24-2-5p), cardiomyogenesis (via miR-195, -223, -208-3p, and -499a), and anti-apoptosis (miR-181b-3p, -150-5p, -302a-5p, and -92a-5p) to the site of injury (Fig. 2) [60]. The exact mechanism of the privileged mobilization of RACs into regenerating areas is not yet clarified, and further in vitro, in vivo, and clinical trials are needed to speculate on the underlying mechanisms for RACs’ preferential recruitment. Also, future investigations are required to address some main aspects: (i) finding the optimum dose, (ii) timetable of transplantation, and (iii) evaluating allogeneic transplantation in small and large animal models of MI.

## RACs therapy for stroke

Stroke conditions are the main reason for physical disability in adults. Some strategies, such as thrombolytic therapy, have been identified to improve stroke survival and disability rates [61]. The high global incidence rate of stroke has energized scientists to develop regenerative strategies to reduce clinical defects caused by ischemic stroke [62]. Recent advances have proposed that cell therapy in conjunction with the conditioning system is more effective in the stroke regeneration process. In this regard, it could be stated that the VCC was established in order to obtain EPCs with high angiogenesis, anti-inflammatory properties, and tissue regeneration in ischemic stroke sites. EPCs, as a fortified fraction of RACs, are able to penetrate the blood–brain barrier to induce angiogenesis and stimulate the retrieval of cerebral ischemic damage [63, 64]. The anti-inflammatory and immune-modulatory cytokines resulting from VCC will cover the antagonistic effects of VEGF at the initial stage of cerebral ischemia [65]. Besides, it is worth noting that intra-arterial RACs transplantation enhanced cerebral blood supply in mouse models of constant middle cerebral artery occlusion and increased VEGF-positive cells in the peri-infarct zone, compared to the placebo-treated group [66]. These findings depict that RACs accelerate the regeneration of neurovascular networks and are considered the ideal strategy for clinical trials against ischemic stroke problems. The amount of newly produced blood vasculature in the penumbra region was meaningfully improved in the RACs group compared to the vehicle-injected group, indicating the strong angiogenic capability of RACs. Experimental findings confirmed that intra-arterial injection with RACs reduced the infarct lesion one day after the occlusion of the middle cerebral artery. Furthermore, VEGF and IL-10, which are important factors for angiogenesis, were significantly increased on the 7th day after the onset of the infarction in the RACs group compared to the control group. Based on these findings, RACs might improve the restoration and redevelopment of neurovascular networks after an acute focal ischemic stroke [65, 66].

The inflammatory cascade and vasculogenesis are the landmarks of the ischemic peri-infarcted zone in the acute stage of cerebral ischemia, which initiate tissue regeneration. Nevertheless, excessive inflammatory responses in acute ischemia inhibit vasculogenesis and tissue regeneration, resulting in tissue destruction [67]. Brain ischemic conditions initiate the secretion of damage-associated factors such as high mobility group protein B1 (HMGB1), heat-shock proteins (HSPs), S100 families, heparan sulfate, and nucleic acids [68]. These factors activate an inflammatory response, leading to the infiltration of immune cells into the brain parenchyma [69]. Because RACs overwhelm neuroinflammation through IL-10 overexpression, it is reasonable to expect that RAC transplantation following cerebral ischemic incidence will result in promising outcomes [70]. Several conceivable molecular mechanisms of neuroprotection and vasculogenesis by RAC could be imagined. RACs elevate the IL-10- and VEGF-positive cell fraction after transplantation [66, 71]. Previously published scientific reports authorized the hypothesis that IL-10 cytokines have a critical function in EPC-associated vasculogenesis [72, 73]. This hypothesis is conceivable that implantation of RACs would participate in microenvironment modulation to stimulate IL-10-mediated anti-inflammatory reactions and VEGF-mediated angiogenesis in ischemic sites of the brain, even though more studies are needed to authorize this declaration. As mentioned above, RACs initiate anti-inflammatory reactions by producing immune modulators such as IL-10 and VEGF, so that RACs may also decrease the infarct size in human stroke ischemia. RACs are available for clinical use in 7 days, allowing these cells to be transplanted in the subacute stage.

## RACs therapy for peripheral arterial disease

Peripheral arterial disease (PAD) is one of the major health burdens in modern society. Most patients suffering from diabetes mellitus type II undergo a severe PAD complication, i.e., chronic limb-threatening ischemia (CLTI) [74]. The main therapeutic approach for CLTI is surgical endovascular revascularization in order to provide blood supply to the arterial vessel-occluded areas. However, around 20–30% of individuals with CLTI are unable to receive vascular techniques, and amputation appears to be the last option. With severe obstruction of blood flow, type II diabetic patients often have limb loss [75]. Recently, cell-based therapy for treating CLTI has been introduced as a safe and effective promising strategy for treating ischemic tissue and preventing major amputations. [76, 77]. Tanaka et al. evaluated the efficacy of RACs versus G-CSF-mobilized CD34^+^ and early EPCs in a mouse hind limb ischemia model [78]. After RAC transplantation, lased doppler blood perfusion recovery in ischemic hind limbs is superior to early EPC and CD34^+^. Histological evaluations and real-time PCR assays in ischemic hindlimbs demonstrated that RACs enhanced vasculogenesis, myogenesis, and decreased inflammation and fibrosis (41). Tanaka et al. initiated phase I/II clinical trials of RACs therapy on PAD patients and diabetic patients with chronic non-healing ulcers [79]. The treatment procedure for CLTI injuries was significantly accelerated in all 7 patients enrolled in the study by RACs injection, which significantly increased vascular perfusion and skin perfusion pressure and decreased the visual analog scale in all subjects' post-therapy. The findings of this prospective study define immunity and the potential of RAC-based regenerative medicine in ischemic tissue injuries. It was shown that wound healing procedures of all 9 patients enrolled in study have significantly accelerated by RACs injection which significantly increased vascular perfusion, skin perfusion pressure (SPP), TcPO2 and decreased visual analog scale (VAS) scale and pain scale in all subjects’ post-therapy. Besides, from cellular viewpoint, RAC therapy increased population of CD34^+^, CD133^+^, CD206^+^ and CD3^+^ while decreasing the population of CD14^+^ and CCR2^+^ cells. The findings of this prospective investigation specify the immunity and possibility of RAC-based regenerative medicine in diabetic ulcers [80]. This strategy allows scientists and clinicians to implant extremely angiogenic EPCs from small blood samples that can be the earliest safe and curative vascular cell therapy for diabetic ulcers. Taking together, RAC therapy is safe and feasible for patients with PAD and future studies warrant evaluating the dose and transplantation route.

## RACs therapy for ischemic wound healing

Abnormal wound healing is an important healthcare issue, exposing burdens to diabetic patients and the healthcare system. Present treatments are moderately curative and frequently fail to the wound closure, especially in populations with comorbidities such as aging and metabolic disorders. Stem cell-based regenerative medicine has emerged as a promising treatment strategy for improving wound closure by altering immune responses, stimulating vasculogenesis, and restoring tissue to its pre-damaged state [81]. Emerging scientific reports propose that EPC dysfunction in diabetic mellitus causes poor angiogenesis and defective wound healing [82]. As reported previously, VCC could restore EPC functionality and show favorable wound-healing features for diabetic ulcers [49]. Importantly, RACs exhibit drastically enhanced wound closure in diabetic mouse models [55]. Because of the increasing fraction of EPCs in the VCC system, these cells have great potency to attach to the internal surface of the vasculature, migrate, differentiate into endothelial cells (ECs), and participate in angiogenesis [83]. In addition, Tanaka et al. also authorized that RACs could promote wound granulation and maturation as well as vasculature spurting [84]. To further validation, Kado et al. reported in a porcine model that RAC therapy accelerated the production of granulation tissue, augmenting collagen synthesis and maturation, epithelialization, and improved vasculogenesis compared to the control group. In addition to their direct involvement in vasculogenesis, they demonstrated that RACs cause overexpression of paracrine mediators known to be involved in wound healing, such as FGF, vascular endothelial growth factors (VEGFs), IL-4, IL-6, and IL-10 [85], which was supported by Tanaka et al. in RACs-treated wound healing in a murine model [86]. Transplantation of RACs into diabetic ulcers positively modulates anti- and pro-inflammatory elements, growth factors, and cytokines at the wound site, resulting in a shift from an inflammatory to an anti-inflammatory status in the wound microenvironment [85]. Furthermore, after RACs transplantation, TGF-β is significantly secreted in the injured tissues [87]. High TGF-β levels have been shown to improve keratinocyte and fibroblast migration and proliferation, as well as the granulation and wound closure processes [88]. Recently, it has been reported that the levels of the peroxisome proliferator-activated receptor gamma coactivator 1 alpha (PGC-1α) and Notch signaling-related elements were significantly decreased post-RAC therapy, proposing that the reduction of PGC-1 α and Notch proteins have a key function in retrieving the angiogenic potential [55]. PGC-1α is a transcriptional co-activator that could inhibit endothelial migration and angiogenesis, leading to the unresponsiveness of ECs to vasculogenic factors [89]. In line with this, it is well-documented that PGC-1α also prompts Notch signaling cascades and modifies underlying cascades that are essential in the vasculogenesis and migration of ECs [90]. To confirm these findings and reveal the mechanism in a clinical trial setting, Tanaka et al. illustrated that autologous transplantation of PB derived QQ-CD34^+^ considerably enhanced wound closure, re-epithelialization, and reendothelialization in refractory diabetic wounds through increased differentiation ability of diabetic CD34^+^ cells, direct vasculogenesis, and improved expression of angiogenic- and wound curing-related agents, overwhelming the intrinsic inadequacy of autologous cell therapy in diabetic subjects [55]. Additional experiments regarding the underlying mechanisms simplify the policy of RAC therapy for diabetic wound healing and other ischemic conditions. As a more general note, RAC therapy improves tissue regeneration through paracrine secretion of vasculogenic and anti-inflammatory factors, causing a shift from an inflammatory to an anti-inflammatory status in the injury microenvironment.

## RACs therapy for ischemia/reperfusion kidney injury

Renal ischemia/reperfusion damage, which results in unexpected failure of renal function, is one of the major global healthcare concerns due to its increasing prevalence [91]. There is lack of effective treatment of acute kidney injury and current therapies support renal function via dialysis. Stem cell-associated regenerative medicine is a novel and promising treatment for renal ischemia–reperfusion conditions, which could attenuate ischemic injury and accelerate the regeneration process, as validated in many preclinical/clinical investigations [92]. Ohtake et al. have begun to investigate EPC-enriching VCC approaches for the regeneration of acute kidney injuries (AKI). They reported that RACs significantly upgraded renal function and decreased histopathological injury in a mouse model of AKI. Also, authors reported that RACs therapy significantly increased renal bloodstream and decreased blood urea nitrogen and serum creatinine levels after 2 days of injection, leading to protection from vasoconstriction/endothelial damage [53]. It could be postulated that preservation from continuous narrowing of blood vessels, called vasoconstriction, and endothelial impairment are imaginable mechanisms of RACs therapy in AKI model. Moreover, RACs lead to a significant reduction of peritubular capillary loss and interstitial fibrosis in the restoration period [53]. These outcomes may encourage the clinical implementation of RACs therapy for AKI subjects. Clinical application of RACs in AKI is just beginning, and the underlying molecular signaling of its regeneration properties is poorly understood. Additional experiments are therefore obligatory to elucidate the molecular mechanism influenced by RAC preconditioning. One possible mechanism is that trans-differentiation of transplanted cells into tissue-specific cells may have a critical function in the obtained positive findings. Another possibility is that RACs, through paracrine activities, mediate renal functionality repair. The regenerative paracrine activity of transplanted RACs may be correlated to the mechanisms that decrease tissue injury and enhance kidney functions. As above noted, RACs provide vasculogenic (increased number of CD34^+^/CD133^+^ cells) and anti-inflammatory (CD206^+^ cells) conditions in the target tissues. On the other hand, it has been demonstrated that a small fraction of RACs express CD31 markers. As a result, it is possible to conclude that a number of transplanted RACs collaborate with local ECs to retrieve peritubular capillaries injured during the AKI model's recovery phase. According to these outcomes, as a more general note, it could be mentioned that RACs could ameliorate severe AKI in mice and may be a fundamental step to opening the therapeutic window of RAC-based therapy to clinical applications.

## RAC therapy for other diseases

### RACs therapy for bone disorders

Treating bone disease with current therapeutic strategies presents a huge problem for orthopedics and regularly yields disappointing outcomes. Critical size bone defects, aneurysmal cysts, enchondroma, pseudarthrosis, osteonecrosis, and finally insufficient bone regeneration are a small part of these problems. To overcome these consequences, it has previously been well documented that cell therapy methods have progressively positive outcomes for bone regeneration and successful bone healing [93, 94]. Preclinical study conducted by Mifuji and coworkers revealed that RACs increased cells' vasculogenic and anti-inflammatory capacities and could be a favorable option for non-union bone fracture healing [95]. They validated that the osteogenic potency of MSCs is induced by the VCC method. In addition to angiogenesis, their findings propose that CD34^+^ cells, as a part of RACs, could differentiate into osteoblast cells and participate in bone fracture healing [95]. One possible underlying mechanism is elucidated by Kawakami et al. [96], who reported that the SDF‐1/CXCR4 axis in EPCs is a central signaling pathway for bone fracture regeneration in the mode of CXCR4 knockout mice. Furthermore, the induction of the SDF‐1/CXCR4 axis of EPCs results in the hastening of fracture repair [96]. However, the entire set of involved mechanisms must be investigated. It has been reported that M2 macrophages improve the bone fracture healing process [97]. Based on these results, it could be noted that the supplementation of M2 macrophages in RACs has a critical function in enhancing fracture restoration. As a consequence, RACs transplantation is more operative than naive MNCs in improving nonunion bone fracture healing in a rat model [95].

Bisphosphonate-related osteonecrosis of the jaw (BRONJ), another bone-related disorder, is an uncommonly severe adverse effect of bisphosphonates [98]. It is worth noting that EPCs play a key role in the treatment and management of BRONJ conditions [99]. Because of some complexity in EPC isolation and a decrease in the number of these cells with aging [100] and systemic diseases [101], Kuroshima et al. developed RACs for the regeneration purpose of BRONJ. They confirmed that systemic implantation of RACs decreases BRONJ-like injuries via inducing the synthesis of type I collagen, enhancing angiogenesis, decreasing polymorphonuclear cell infiltration, suppressing the inflammatory response, and increasing anti-inflammatory macrophages in the connective tissue of the tooth extraction area, and consequently accelerating wound healing. Furthermore, RAC transplantation partially recovered the osseous curing process with an increase of active bone, a decrease of necrotic bone, and positively altered bone parameters in the mouse model [102]. It has been documented that RAC therapy sponsored osseous healing with the induction of angiogenesis in bone fracture zones [95]. Aside from inducing angiogenesis, RACs therapy significantly restored the osteoclast cell population, contributing to partial bone recovery. Moreover, RAC therapy is more effective than EPC therapy, suggesting that implantation of a few RACs rather than EPCs might stimulate macrophage migratory potential and new vascularization in injured tissues. These advantages of RAC treatment in bone regeneration medicine may be able to alleviate several challenges with cell therapies, such as cost-effectiveness, safety concerns, the culture time, moral questions, and unfavorable transplantation outcomes. However, the road is long, and we are at the beginning of this road.

### RACs therapy for irradiated salivary gland

Stem cell-based therapies could be a potential approach for treating these conditions and decreasing radiation-induced hyposalivation and other side effects of radiotherapy and chemotherapy [103]. In the previously published report, Takashi et al*.* declared that a stem cell therapy procedure based on VCC has beneficial effects on radiogenic salivary hypofunction. They demonstrated that: (i) treatment by RACs efficiently recovered saliva secretion; (ii) RACs obviously supported tissue regenerative processes in the atrophic salivary glands through their anti-inflammatory and angiogenesis functions; and (iii) RACs probably affected these outcomes via paracrine activities or vascular differentiation [104]. Furthermore, RACs aid in the recovery of atrophic salivary glands by increasing EPC fraction and shifting macrophage phenotype toward an anti-inflammatory M2 macrophage via IL-10 and VEGF production [105]. It has been documented that the M2 macrophages are involved in the deactivation of inflammation and fibrosis in damaged tissues [106]. Therefore, it is worth noting that RACs therapy, composed of M2 macrophages, lymphocytes, and EPCs, could be effectively employed for salivary gland malfunction. These findings showed that the RACs approach could be a hopeful option for emerging future therapeutics and more studies are necessary to elucidate the possible mechanisms of atrophic tissue regeneration by transplanted RACs.

## Route of RAC delivery

Emerging reports on cell therapy trials implied that the efficacy of cell therapy was limited by poor engraftment of cells or that engrafted cells disappeared several months after transplantation [107]. Depending on the disease state and location, cell transplantation routes may differ, and it is difficult to find a universal cell administration route due to several obstacles such as engraftment or cell retention, trapping on other organs, and cell biological/functional impairment due to the transplanted tissue microenvironment [108]. Classical intravenous transplantation of cells has been widely used in preclinical and clinical studies, and their retention depends on cell type and is mostly low in ischemic heart diseases [109]. However, depending on the transplanted cell type, several beneficial functions of systemic transplantation are listed, including (i) no requirement for special sophisticated delivery techniques, (ii) the immunomodulatory effect of transplanted cells may benefit the whole organism, and (iii) the option for repetitive transplantation. Sometimes, the desired results cannot be obtained after one transplantation; consequently, repetitive systemic transplantation via the vein is needed, whereas in several diseases, local transplantation is not allowed for this technique [110]. As we demonstrated experimentally, the transplanted RAC cells returned to the site of injury and were incorporated with heart and brain cells, indicating superior potential to overcome trapping in the lung and other organs. The local RAC delivery method is promising in terms of chronic ulcer size reduction, edema decrease, and acceleration of granulation signs [55]. These macroscopic findings were reported 2–4 weeks after local delivery of RACs. The possible beneficial effect of local ulcer size improvement couple with paracrine effect of RAC such as extracellular vesicles-derived miR and interleukins and growth factors. For target organ delivery, it has been shown that the local tissue inflammatory environment and activation of receptors and ligands (adhesion molecules and chemokines) play essential roles in cell uptake. Further studies are required to elucidate the mechanism of RAC infiltration at the site of injury. This information is valuable for the in vivo biodistribution of RACs and the control of dose and potential side effects.

## Conclusion

**Fig. 3:**
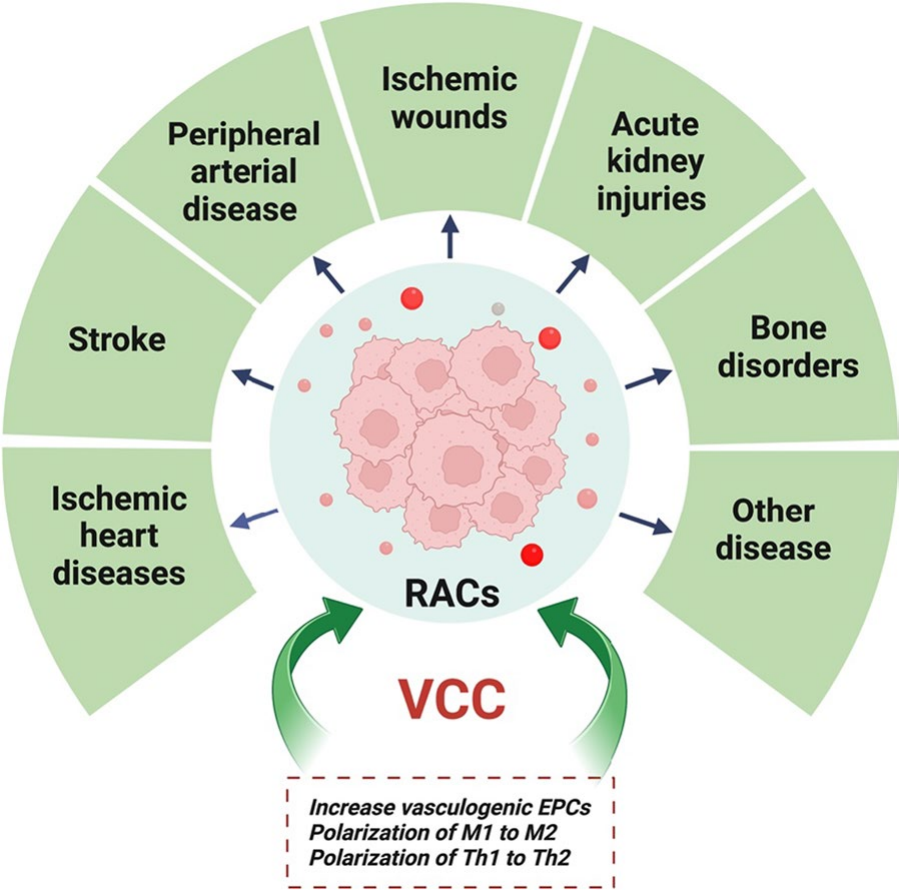
Regeneration-associated cells application. Figure created with BioRender.com

Microenvironment therapy is required for regenerative medicine, and it is worth noting that RACs, including EPCs, M2 macrophages, and Tregs, are a new tool for regenerative microenvironment therapy. At a glance, the current study introduces the VCC and demonstrates its therapeutic efficacy in a variety of diseases. Angiogenic, anti-inflammatory, immune-suppression, immune-tolerance, and wound-healing effects of RACs were validated in in vitro, in vivo, and clinical trial models. This method addresses the inadequate effectiveness of the current EPC therapy for therapeutic goals in several diseases. This study, also demonstrates that the *ex vivo* conditioning of MNCs as RACs is a novel and encouraging therapeutic opportunity for patients with AMI and other vasculogenic-related disorders (Fig. 3). Further experiments on the fundamental mechanisms could assist in the design of more efficient therapeutic modifications for different disorders. If repeated positively in several clinical trials, the RAC system could obtain a cellular product that will efficiently regenerate ischemic and other diseases in humans, achieving the principal goal of regenerative medicine research. Further studies are required to address the optimal cell treatment dose for each acute or chronic stage of disease. It is worth noting that repeated transplantation of RAC-derived EVs was beneficial in curing myocardial infarction; however, the benefits of repeated versus single-shot RAC transplantation must be determined.

## Data Availability

Not applicable.

## Abbreviations

AKI: Acute kidney injuries
BM: Bone marrow
BRONJ: Bisphosphonate-related osteonecrosis of the jaw
CLTI: Chronic limb-threatening ischemia
CPCs: Cardiac progenitor cells
DC: Dendritic cells
ECFCs: Endothelial colony-forming cells
ECs: Endothelial cells
EPCs: Endothelial progenitor cells
HMGB1: High mobility group protein B1
HSPCs: Hematopoietic stem and progenitor cells
HSPs: Heat-shock proteins
IL-1: Interleukin-1
INF-γ: Interferon-gamma
iNOS: Inducible nitric oxide synthase
MHC: Major histocompatibility complex
MIP-1α: Macrophage inflammatory protein-1α
MMP-9: Matrix metallo proteinase-9
MNCs: Mono nuclear cells
MaSC-Evs: MSC-derived extracellular vesicles
MaSCs: Mesenchymal stem cells
PAD: Peripheral arterial disease
PB: Peripheral blood
PD-L1: Programmed death-ligand 1
PGC-1α: Peroxisome proliferator-activated receptor gamma coactivator 1 alpha
PGE2: Prostaglandin E2
RAC-EVs: RAC-derived extracellular vesicles
RACs: Regeneration-associated cells
SPP: Skin perfusion pressure
TAC: Tumor-associated cells
TAM: Tumor-associated macrophages
TAN: Tumor-associated neutrophils
TGF-β: Transforming growth factor beta
VAS: Visual Analog Scale
VCC: Vasculogenic culture conditioning
VEGFs: Vascular endothelial growth factors
